# YOLOv8-CML: a lightweight target detection method for color-changing melon ripening in intelligent agriculture

**DOI:** 10.1038/s41598-024-65293-w

**Published:** 2024-06-22

**Authors:** Guojun Chen, Yongjie Hou, Tao Cui, Huihui Li, Fengyang Shangguan, Lei Cao

**Affiliations:** 1 Qingdao Institute of Software, College of Computer Science and Technology, China University of Petroleum (East China), Qingdao, 266580 China; 2 College of Information Science and Technology, Qingdao University of Science and Technology, Qingdao, 266100 China; 3 Faculty of Light Industry, Qilu University of Technology, Jinan, 250300 China; 4 State Key Laboratory of Biobased Material and Green Papermaking, Shandong Academy of Sciences, Jinan, 250300 China

**Keywords:** Attention mechanisms, Color-changing melon dataset, Intelligent agriculture, Target detection, YOLOv8n, Image processing, Machine learning

## Abstract

Color-changing melon is an ornamental and edible fruit. Aiming at the problems of slow detection speed and high deployment cost for Color-changing melon in intelligent agriculture equipment, this study proposes a lightweight detection model YOLOv8-CML.Firstly, a lightweight Faster-Block is introduced to reduce the number of memory accesses while reducing redundant computation, and a lighter C2f structure is obtained. Then, the lightweight C2f module fusing EMA module is constructed in Backbone to collect multi-scale spatial information more efficiently and reduce the interference of complex background on the recognition effect. Next, the idea of shared parameters is utilized to redesign the detection head to simplify the model further. Finally, the α-IoU loss function is adopted better to measure the overlap between the predicted and real frames using the α hyperparameter, improving the recognition accuracy. The experimental results show that compared to the YOLOv8n model, the parametric and computational ratios of the improved YOLOv8-CML model decreased by 42.9% and 51.8%, respectively. In addition, the model size is only 3.7 MB, and the inference speed is improved by 6.9%, while mAP@0.5, accuracy, and FPS are also improved. Our proposed model provides a vital reference for deploying Color-changing melon picking robots.

Color-changing melon, scientific name Trichosanthes cucumerina L. (Sp. Pl. 2: 1008. 1753), is an annual herbaceous plant within the Cucurbitaceae family. Native to India and Malaysia, it is highly productive and adaptable and is grown in both southern and northern China. Color-changing melon has a fruiting period of up to four months, and the fruits ripen inconsistently, leading to a major reliance on human labor in the current harvesting process. Early or late harvesting affects fruit quality and farmers' profitability^1^. As a result, multiple harvesting by hand is currently required, which entails considerable time and labor expenditures^2^ and inefficient in production. In order to solve these problems in intelligent agriculture, researchers consider the development of intelligent robots for fruit inspection and robotic picking^3^ as an effective approach. This is because automated robotic picking can reduce farmers' labor intensity, improve picking efficiency and quality, shorten the picking cycle, and save labor costs. Generally speaking, the main working parts of the robot are the robotic arm and embedded devices. The robotic arm is responsible for picking fruits, the detection camera is responsible for positioning and classifying the fruits, and the embedded microcomputer judges the image, thus controlling the trajectory of the robotic arm. However, robotic harvesting also requires solving some technical challenges, a key step of which is how to achieve fast and accurate target detection for Color-changing melons.

Currently, the picking and harvesting of fruits in the farmland relies mainly on manual labor, but as the trend of rural young people migrating to the cities becomes more and more obvious, the decline of the proportion of working-age people and the rise of labor costs have become a non-negligible problem^4^. However, with the expanding application areas of deep learning^5^ and the trend of agriculture toward intelligent agriculture^6^, image processing has become an significant research direction in the field of agriculture. Artificial intelligence methods for target classification, disease detection, and fruit localization, among others, have been heavily researched in agricultural practices^7^, which has led to the promise of automated harvesting robots as an alternative to human manual labor^8^. Using automated harvesting robots in the fruit harvesting process can significantly lower labor costs and alleviate labor scarcity in the centralized harvesting process of agricultural products, especially in the case of fruit harvesting that meets the conditions for mechanization^9^. However, in natural environments, the interference of external factors such as light variations, fruit overlapping, and background color similarity can affect the results of target detection to some extent^10^. Therefore, accurate detection of target fruits in complicated agricultural environments is an important prerequisite for the realization of automated sorting robots.

Prior to the application of neural networks, researchers used traditional algorithms like edge detection, region growing, threshold analysis, and grayscale covariance matrices to extract features like shape, texture^11^, and color^12^ for the recognition of fruits and their backgrounds. Reference^13^ suggested an OTSU thresholding algorithm that automatically calculates segmentation thresholds while extracting color features in OHTA color space. Reference^14^ used adaptive histogram balancing, OTSU thresholding, and red-blue combination mapping approach to segment the target image. Reference^15^ proposed a method considering color features for citrus recognition. Although the above methods solve the problems encountered during fruit detection to some extent, the feature information obtained by these algorithms needs to be manually pre-designed, and the extracted features are only suitable for specific background conditions, thus lacking sufficient generalization. In addition, for green fruits in natural environments, relying solely on color features can be very limiting and unstable due to the interference of vine manes and leaves^16^. Similarly, for immature Color-changing melons that are similar to the background in color, it is challenging for a picking robot to detect the fruits based on color features^17^. Therefore, to effectively identify Color-changing melons, some other visual information, like texture and shape, is needed in addition to color features. For instance, Ref.^2^ proposed a network model that consists of detail feature enhancement and content-aware reorganization modules for extracting texture features of blueberry fruits, which improves the accuracy of detection.

Several traditional machine-learning approaches have been utilized for fruit detection, for instance, Bayesian classifiers^18^ and support vector machines^19^. However, these traditional methods have limitations in that they are usually unable to learn high-dimensional features directly and thus cannot fulfill the real-time requirements in real production. Nowadays, deep learning-based target recognition algorithms are progressively finding applications within the domain of intelligent agriculture, and these detection algorithms are centered on classification tasks and localization tasks. According to the different methods, these detection algorithms are able to be categorized as single-stage and two-stage. The two-stage generates proposals regions at first and then predicts the classification and localization of targets through neural networks. Representative models of such algorithms are the R-CNN family, such as Fast R-CNN, Faster R-CNN^20^, and Mask R-CNN^21^. Faster R-CNN achieves faster detection^22^. Reference^23^ applied Faster R-CNN to detect various fruits, such as apples and melons. Reference^24^ integrated Faster R-CNN with transfer learning for detecting unripe tomato. On the contrary, representative models of single-stage detection algorithms that perform feature extraction directly through a neural network, i.e., simultaneous detect target classification and localization, are the SSD^25^ and YOLO series^26^. These algorithms have faster detection speeds and are more appropriate for applications in real-time detection situations.

Until today, the YOLO family of models has been rapidly developed and widely used in fruit detection^27–29^. Reference^30^ suggested the Des-YOLOv3 model, which was improved from the original YOLOv3 model^31^ for the recognition of citrus fruits under nocturnal conditions with an accuracy of 90.75%. Compared to the Faster R-CNN model^32,33^, which uses ZFNet and VGG16 as Backbone, the YOLOv3 model shows significant improvement in accuracy and speed of fruit detection^34^. However, the YOLOv3 model suffers from relatively complex network structure and FLOPs.YOLOv4 introduces CSPDarknet53^35^, SPP module, and FPN + PAN structure, improves the loss function, and also uses Mosaic data augmentation to realize the model to obtain a balance between accuracy and speed in fruit detection^36,37^. To address the problem of segmenting instances of healthy and diseased tomato seedlings during the growing period, Ref.^38^ proposed an improved YOLOv8-seg model, which is capable of accurately segmenting tomato plant images in complex backgrounds. Although the above algorithms achieve excellent detection results, they are usually accompanied by large model size, parameters and FLOPs, which can hinder the deployment of the improved model on edge devices. As a result, numerous researchers have also begun to focus on lightweight methods for target recognition algorithms. Reference^39^ used an improved Bottleneck to lower parameters and FLOPs while obtaining features at long distances in space. Reference^40^ suggested an improved cherry tomato ripeness detection algorithm based on YOLOv5, which uses a coordinated attention mechanism and dynamically focused bounding box regression loss. It optimizes the model with an accuracy of 95.2%, and the weight file is 4.4 MB. Reference^41^ used the MobileNetV3 network to replace the original YOLOv5's Backbone and then optimized the hyper-parameters using a genetic algorithm to successfully deploy the model to mobile terminals, and its maximum detection speed is 32.2 FPS.

In summary, Color-changing melon detection algorithms have not yet achieved the ideal balance between speed and accuracy. Much of the previous work has focused on improving the accuracy of the models but has not considered the cost of deployment on resource-constrained devices. In addition, most models consider the impact of complex backgrounds on detection accuracy but do not pay much attention to the detection speed of lightweight models.

In our study, YOLOv8n was chosen as the base model to investigate lightweight algorithms to improve the versatility and speed of color-changing melon detection algorithms on different devices. The main contributions and innovations of this study are summarized as follows:

1. We created a Color-changing melon figure dataset.
2. We proposed a lightweight detection algorithm based on the YOLOv8n model, called YOLOv8-CML. It improves detection speed while keeping the model lightweight and is more suitable for running on resource-constrained devices, which can provide some ideas for subsequent deployment.
3. We designed several comparative experiments for detecting discolored melons in the dataset to illustrate the performance difference between different algorithms and demonstrate the recognition effect of the model.

The experimental results show that YOLOv8-CML exhibits excellent overall performance compared to other lightweight algorithms. Therefore, our algorithm can provide suitable technical support for the robotic picking of Color-changing melons in intelligent agriculture.

## Materials and methods

### Data acquisition and labeling

**Figure 1:**
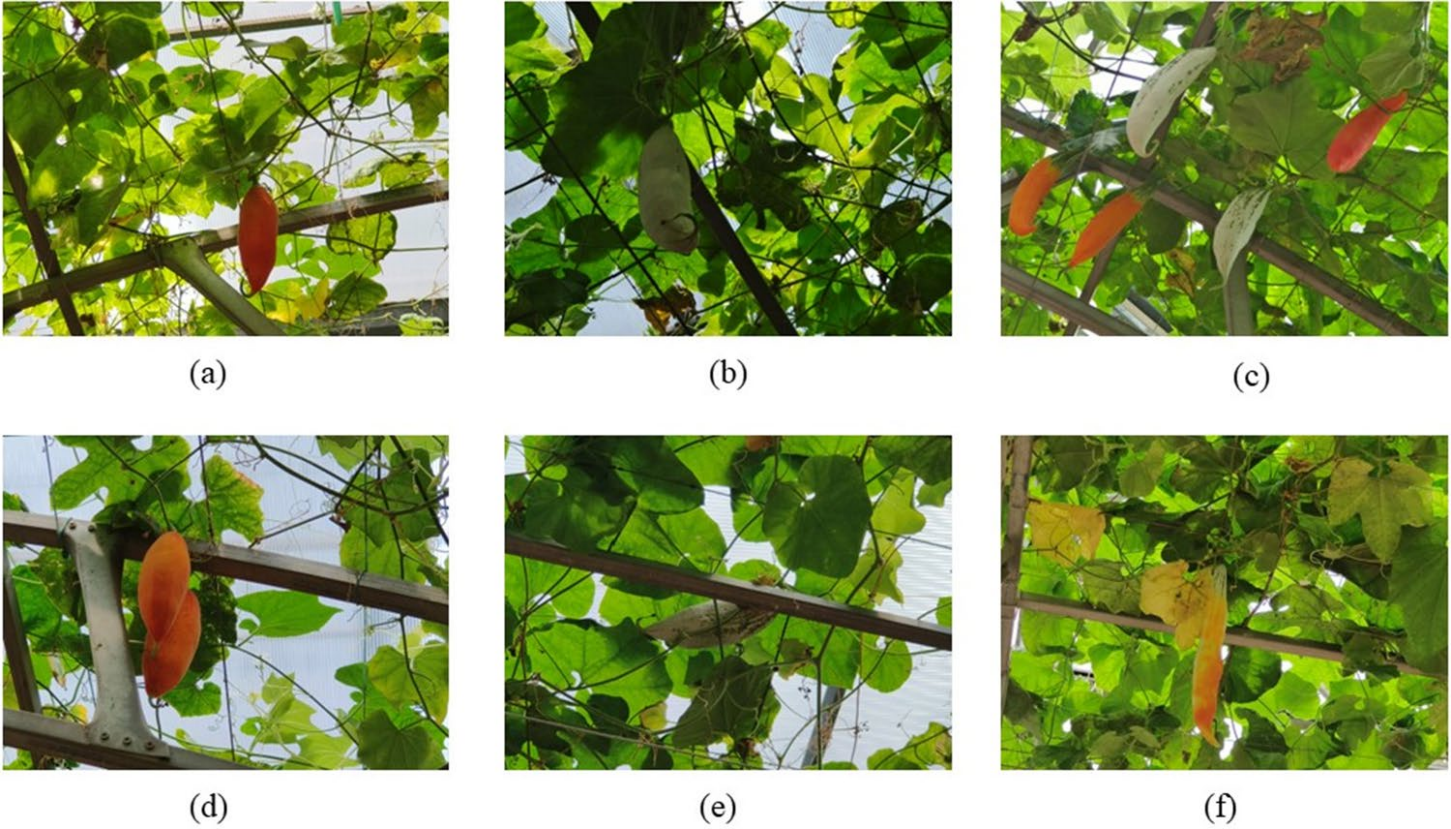
Color-changing melon samples from different scenarios.

Color-changing melon images were captured from Vegetable High-Tech Demonstration Park, No. 108, Luocheng Street, Shouguang City, Weifang City, Shandong Province, China. The location is marked by a latitude of 36.855968 and a longitude of 118.818556. All images were collected in July 2023 at a distance of 80–120 cm using a vivo X80 phone equipped with a Sony IMX 866 RGBW outsole sensor. Owing to the constrained variety within the training samples and to reduce the risk of model overfitting, we captured images from three angles: directly in front, directly behind, and at the bottom of the Color-changing melon. These images include light and dark variations, branch and leaf shadows, and fruit overlap, which are common in the picking process. Figure 1 shows samples from our Color-changing melon dataset obtained in different scenarios. In total, we collected 1297 original Color-changing melon images, which were saved in JPG format with $4032\times 3024$ pixels. We then screened the captured raw images to exclude low-quality images such as over-blurred, overexposed, heavily occluded, and incomplete cases. Finally, we generated a dataset containing 1240 Color-changing melon images.

Color-changing melon (scientific name Trichosanthes cucumerina) is a monoecious, heterogamous plant with a fruiting period of up to four months. A plant can simultaneously yield fruits of varying degrees of maturity, comprising mature, semi-mature, and immature fruits, depending on when the female flowers are pollinated. The exterior color of Color-changing melons gradually changes from green to yellow and eventually to red as maturity increases. The key indicator of ripeness in Color-changing melons is the color of the rind surface. Color-changing melons at the immature stage usually have light or dark green rinds, and some fruits may have finely striped rinds, which make them suitable for eating. As the ripening process progresses, the melon rind color gradually changes from green to orange, a change that extends from the base of the melon to the vines, marking the beginning of the semi-mature stage. By the time it reaches the mature stage, Color-changing melons have a deep red color on their surface, and usually, when these fruits are picked, the seeds are removed for next year's planting. In practical production applications, we need to pick different types of fruits in the same season due to different uses. Using algorithms to acquire information about fruits helps to enable precise location and classification, which in turn offers a reference for the work of automated harvesting. In this study, green rinds were considered to represent immature Color-changing melons, while rinds with some or all of their surface color orange were considered to be semi-mature Color-changing melons, and dark red rinds represented mature Color-changing melons. In order not to affect the extraction of Color-changing melon features and to reduce the computational load, the dataset containing 1240 Color-changing melon images was first compressed to a resolution of $640\times 640$ pixels and then randomly split into training set, validation set, and test set on the scale of 8:1:1 so that there are 992 images in the training set, 124 images in the validation set, and 124 images in the test set. Next, the images are labeled utilizing the LabelImg image annotation tool. For each image, the labels are stored in PASCAL VOC format and YOLO format to suit different algorithms.

### Data augmentor

**Figure 2:**
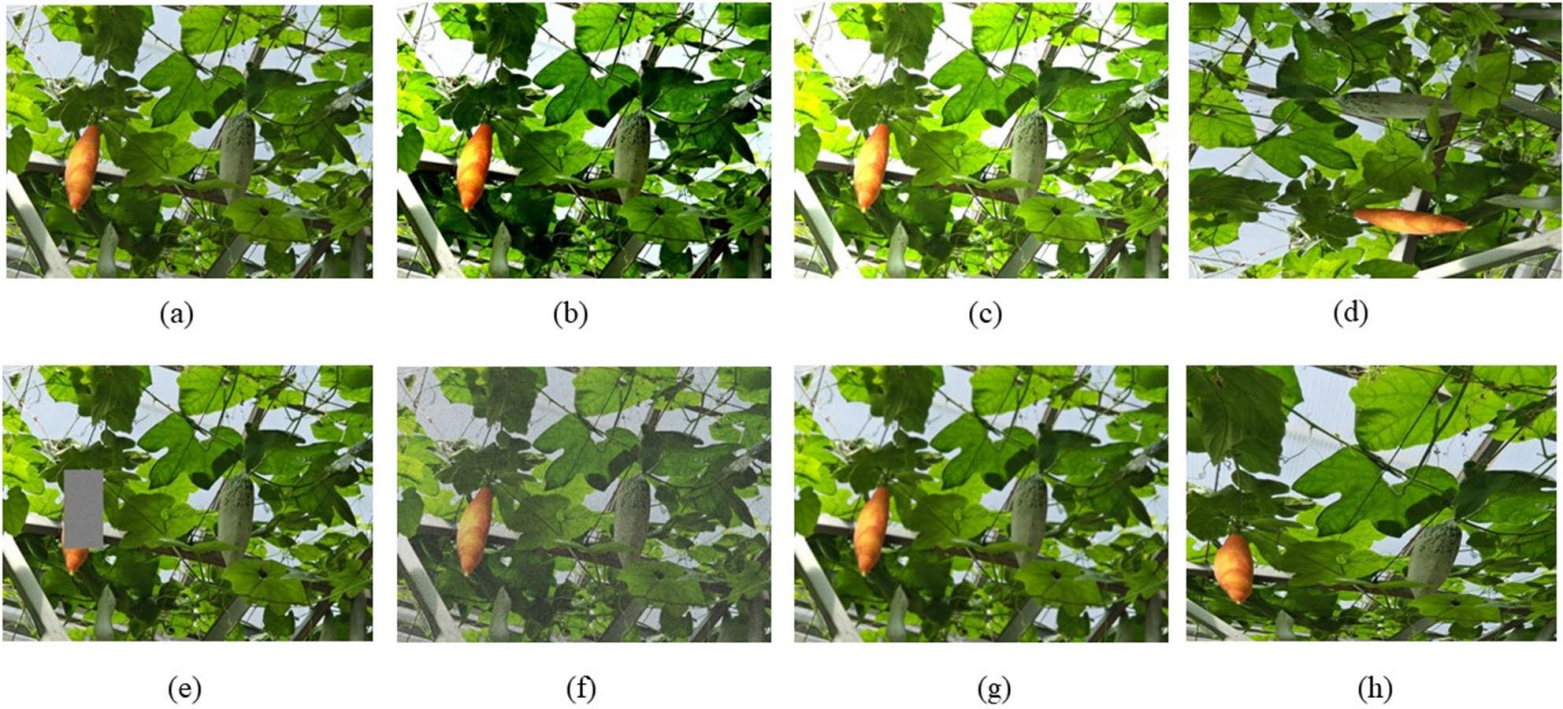
Image augmentation results.

After completing the Color-changing melon image annotation, we apply the Augmentor tool to extend the training set to augment the fruit features, avoid overfitting, and improve the generalization of the model. The data augmentation approaches comprise adjusting horizontal rotation, left and right mirroring, vertical mirroring, brightness, contrast, random distortion, Gaussian noise, and Gaussian blur. Table 1 provides a description of all enhancement operations, while some enhanced images are shown in Fig. 2. After data augmentation, the images in the training set are expanded from the original 992 to 2142. The number of labels for each set of three categories is shown in Table 2.

**Table 1:** Number of Color-changing melon images under various data augmentation techniques.

Operation	Description	Number of images
Rotation	Rotate 90° and 270°	150
Brightness	Enhance and reduce brightness	150
Mirror	Horizontal and vertical mirroring	100
Contrast	Enhance and reduce contrast	150
Erasure	Randomly erase	150
Deformation	Randomly deformed	150
Noise	Add Gaussian noise	150
Blur	Add Gaussian blur	150

**Table 2:** Number of labels per set of three categories.

Category	Immature	Mature	Semi-mature	Total
Train	2147	1178	899	4224
Validation	726	395	307	1428
Test	462	273	208	943

### YOLOv8-CML model

**Figure 3:**
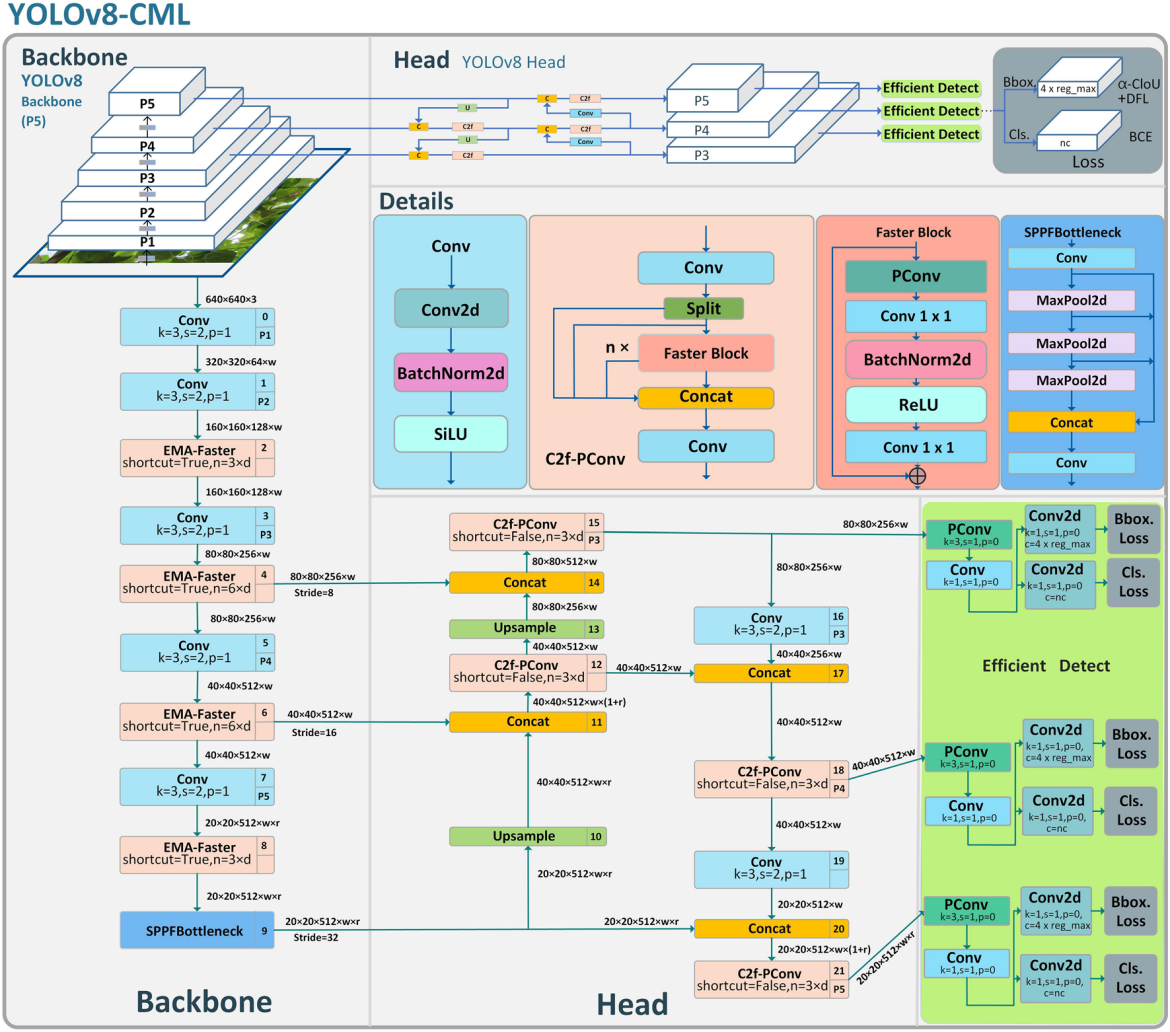
YOLOv8-CML model structure.

With the wide extension of computer vision in several fields, target detection algorithms centered on Convolutional Neural Networks (CNNs) are playing an progressively important role in modern agriculture. Among these algorithms, YOLOv8 is a deep learning-based real-time target detection and image segmentation model released in January 2023 by Ultralytics. For now, YOLOv8 is the newest version of the YOLO family, which improves its comprehensive performance compared to previous versions while providing a unified framework for model training. In addition, it is suitable for an extensive range of target detection, target tracking, instance segmentation, image classification, and so on. The model structure of YOLOv8 is divided into three main parts: (1) Backbone for extracting target features; (2) Neck for fusing multi-scale feature information; (3) Detect head for predicting confidence, category, and anchor frames. YOLOv8 is inspired by VGG, and Backbone references the network structure of CSPDarkNet-53, with the count of channels doubled after the pooling operation. To improve accuracy, Mosaic data augmentation was disabled for the last ten rounds before the end of training. The neck adopts the CSP idea, which fuses the original feature part and the feature part processed by multiple Conv operations to improve feature extraction. YOLOv8 refers to the design idea of ELAN in YOLOv7, and both Backbone and Neck adopt the C2f structure, which makes the feature information richer. The head utilizes the decoupled head structure in the current mainstream, detaching the classification head from the detection head, and the classification branch continues to use the BCE (binary cross entropy) loss, and the regression branch contains the distribution focus loss^42^, and adopts the idea of Anchor Free. Like YOLOv5, YOLOv8 provides models in n, s, m, l, and x scales based on scaling factors to suit different scenarios. YOLOv8n is the smallest model in the YOLOv8 family, which features fewer parameters and lower hardware requirements. In this study, YOLOv8n is chosen as the foundation model to lower computing and memory expenses and to lighten up the model, thus laying the foundation for deployment on embedded or mobile devices in real-time target detection situations^42^. Therefore, we present a YOLOv8-CML model, which is based on the YOLOv8n model with the goal of increasing the detection speed while maintaining smaller parameters and FLOPs. The structure of the improved model is shown in Fig.3.

### C2f-PConv module

**Figure 4:**
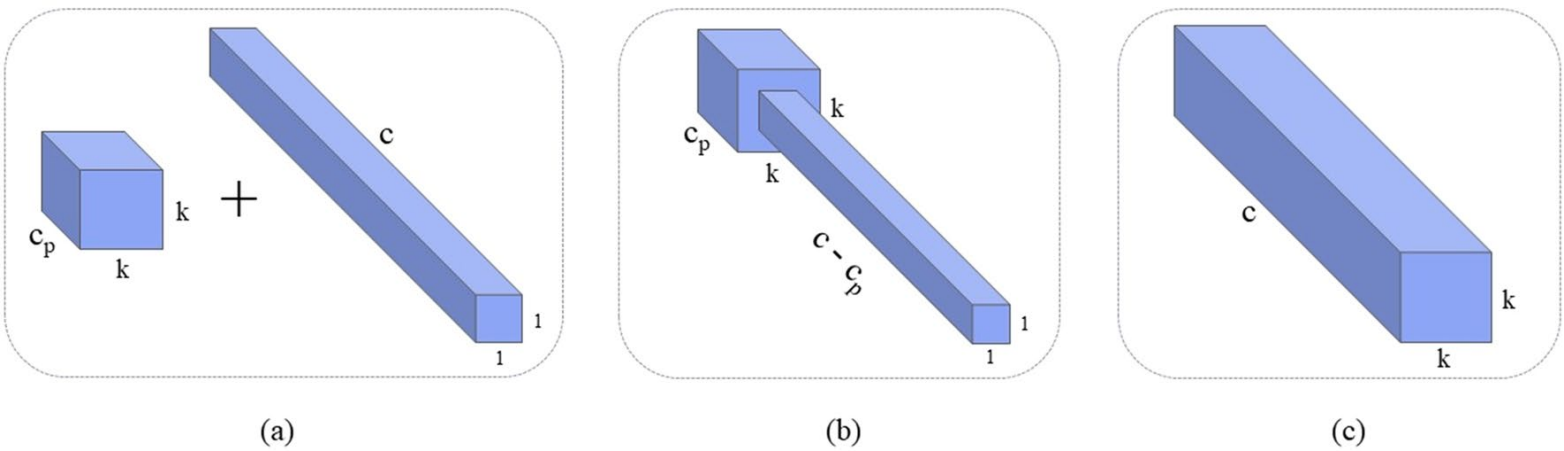
PConv and PWConv structures.

**Figure 5:**
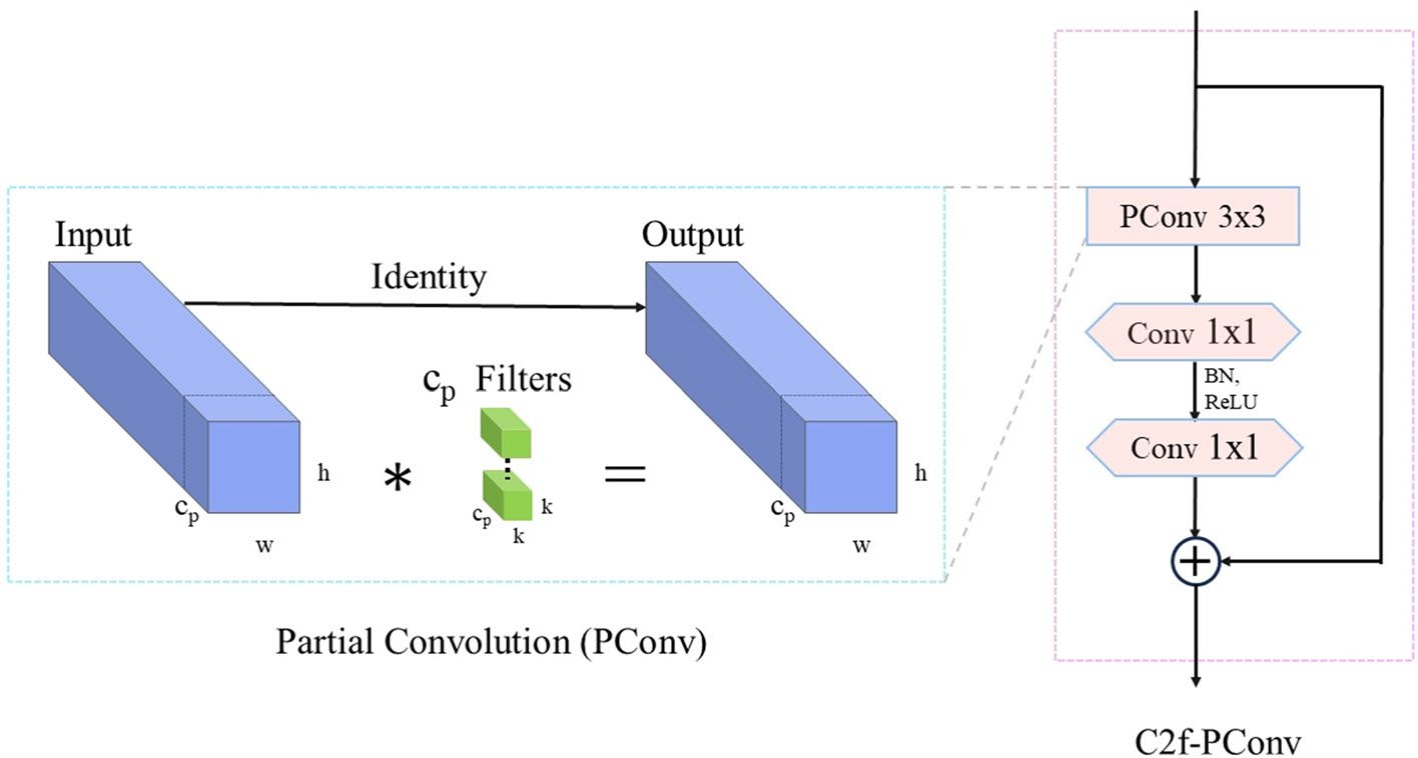
C2f-PConv module.

To optimize the increase in parameters and FLOPs associated with the use of the CSP module^43^ in YOLOv8, we refer to the approach of the FasterNet^44^ model and use the C2f-PConv module in place of the multiple convolutional operations of the CSP module. We introduced the PConv architecture in the C2f module, which, unlike regular convolution, is a partially channel-enhanced Convolution framework that is capable of applying regular Conv for spatial feature extraction from a specific subset of the input channels while keeping the remainder of the channels unaltered. When consecutive or regular memory accesses are required, PConv usually ignores the information of the other channels and uses only the first or last consecutive channel to represent the whole feature mapping, which reduces feature redundancy. In order to utilize the information of each channel more efficiently, point-by-point state convolution (PWConv) is added to PConv, the principle of which is shown in Fig. 4a, which is able to form a T-shaped Conv pattern in the valid sensory field of the input feature map, which is shown in Fig. 4b. Compared with the regular Conv method shown in Fig. 4c, the special pattern of T-shaped Conv emphasizes the importance of the center position more. The left side of Fig. 5 shows the operation of PConv. PConv selectively performs regular Conv operations on the part of the input channels while reserving the rest of the channels for spatial feature extraction. Typically, when consecutive or regular memory accesses are required, PConv chooses the first or last consecutive channel to represent the whole feature mapping.

To accommodate different cases, we assume that an equal count of channels are used to map the input and output features. Given a feature map $F\in {\mathbb{R}}^{h\times w\times c}$, the FLOPs are computed as follows for a $k\times k$ regular Conv:

1 $$\begin{array}{c}FLOP{s}_{Conv}=h\times w\times {k}^{2}\times {c}^{2}\end{array}$$

Assuming that PConv processes only  ${c}_{p}$ channels and the remaining $c-{c}_{p}$ channels are only constant mapped, the FLOPs for PConv are computed as follows:

2 $$\begin{array}{c}FLOP{s}_{PConv}=h\times w\times {k}^{2}\times {c}_{p}^{2}\end{array}$$

Taking the empirical hyperparameter $r=\frac{{c}_{p}}{c}=\frac{1}{4}$, from Eqs. (1) and (2), the computational cost, measured in FLOPs, for PConv, is merely 1/16th of that compared with regular Conv. This is because the Conv operation in this part is the same as the regular Conv operation, and thus PConv, reduces FLOPs by processing only a small portion of the channels while retaining the high FLOP characteristics of the regular Conv.

As shown on the right side of Fig. 5, each C2f-PConv module contains a PConv layer and two Conv layers with kernel size $1\times 1$, which combine to form an entity represented as an inverted residual block, where the second layer contains a larger number of channels. To facilitate the reuse of input elements, a shortcut is created in front of the first layer to recycle the input features. Considering that previous work overused Batch Normalization and activation layers in the model, this can limit the diversity of features and reduce the overall speed of computation^45,46^. Therefore, the C2f-PConv module only uses the Batch Normalization and ReLU activation function behind the second layer to maintain good performance.

### Efficient multi-scale attention

The application of attentional mechanisms in computer vision cannot be ignored. It facilitates the model's learning process by allocating distinct weights to individual sections of the input information. This helps the model to recognize the more significant information in it and improves the performance of the model while mitigating overfitting^47^. Immature color-changing melons exhibit color similarities with canes and leaves. Thus, the differences in shape and texture between the fruit and other background objects become more important. Considering this, using an attention module can effectively add critical information to improve detection accuracy. Efficient Multi-Scale Attention module is an enhanced feature aggregation method^48^, which consolidates specific channels within batch dimensions and partitions the dimensions of channels into several sub-feature groups, and efficiently preserves the channel-specific information. Thus, the spatial semantic features are fairly ensured in the distribution of every feature group.

**Figure 6:**
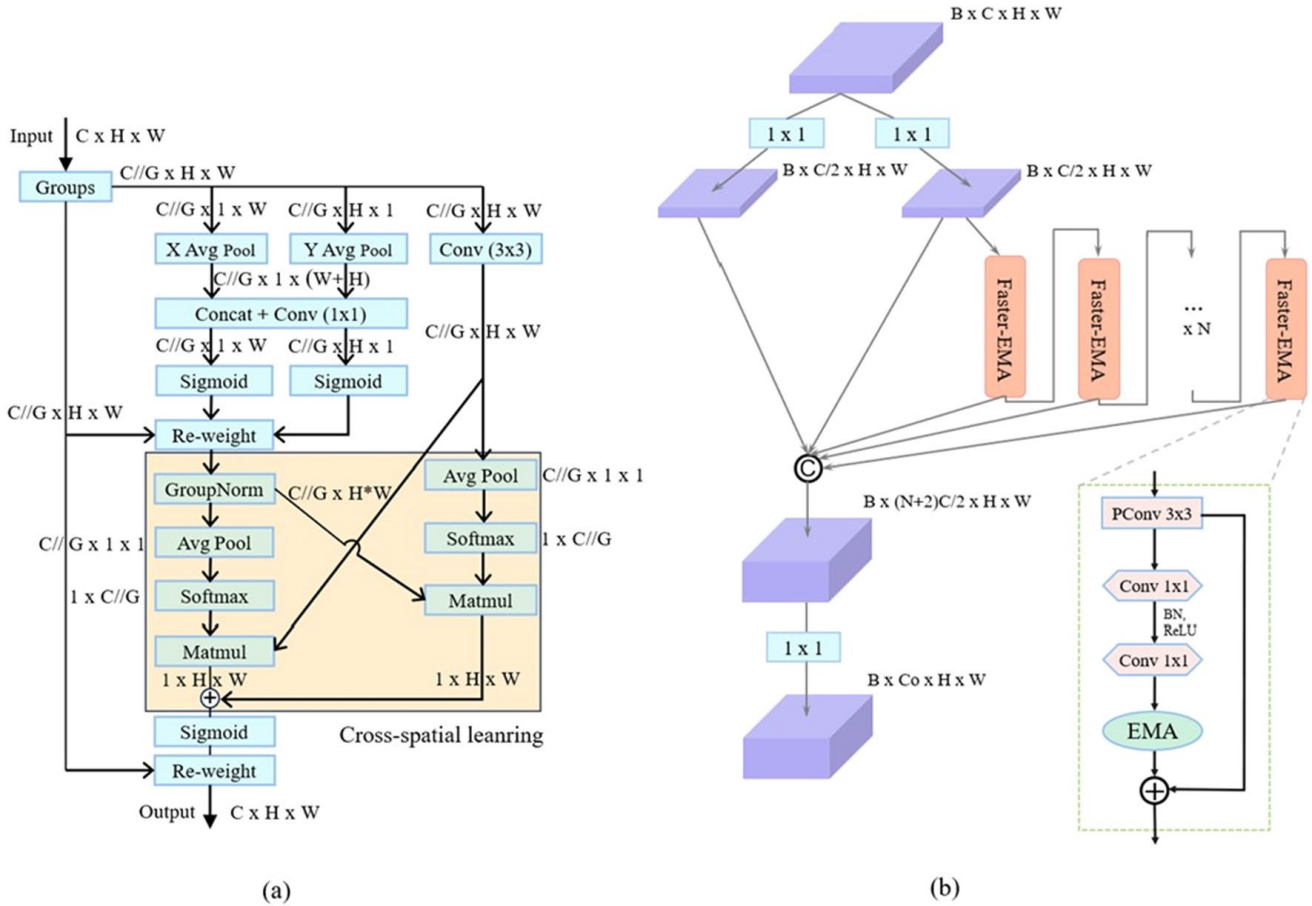
EMA (Efficient Multi-Scale Attention) Block and EMA-Faster structure.

The overall structure of Efficient Multi-Scale Attention is shown in Fig. 6a. The Efficient Multi-Scale Attention mechanism adopts three concurrent paths to fetch the features describing the attention weights in order to group the feature maps. Two paths use $1\times 1$ Conv layers, while the third uses one $3\times 3$ Conv layer. In the $1\times 1$ Conv branch, the channel information is encoded into the global space by a 2D global average pooling operation, and the export of the minimum branch undergoes direct conversion to conform to the type of the relevant dimension before the channel features are united. The function of the $1\times 1$ Conv is to encode the information coming from both spatial directions, enabling the model to interact across channels. In $3\times 3$ branching, the g lobal space information is also encoded using a 2D global average pooling operation. The 2D global pool operation is used to encode the global information extracted from the feature map while modelling the long-range information as dependencies.

The equation of the 2D global pooling operation is expressed as follows:

3 $$\begin{array}{c}{z}_{c}=\frac{1}{H\times W}\sum_{j}^{H} \sum_{i}^{W}{x}_{c}\left(i,j\right)\end{array}$$

where ${z}_{c}$ is the output associated with the c th.

In this study, we add the Efficient Multi-Scale Attention module to C2f-PConv and name it Faster-EMA to replace the C2f module in the Bottleneck section. The improved structure is shown in Fig. 6b. Because the Efficient Multi-Scale Attention module has the ability of cross-space learning, i.e., it has the ability to integrate information from various spatial dimensions, facilitating more overall feature integration. Therefore, replacing the original C2f module with Faster-EMA can extend the depth and acceptable domain of the model and enhance the ability of feature integration to more accurately detect Color-changing melons with different maturity levels. Faster-EMA contains three Conv layers and an attention module. Specifically, the first layer is PConv, which processes only some of the features, and the output channel is equal to the input channel. The second layer is $1\times 1$ Conv, and the count of channels is doubled. The third layer is still $1\times 1$ Conv, but the count of channels is halved. The fourth layer is the Efficient Multi-Scale Attention module, which matches the count of input channels to the count of output channels after Efficient Multi-Scale Attention module processing, according to Fig. 6a. Finally, the performance of the whole module is improved by using shortcut connections. It is worth noting that only the $1\times 1$ Conv of the second layer applies the Batch Normalization and ReLU activation functions.

### Efficient detect

**Figure 7:**
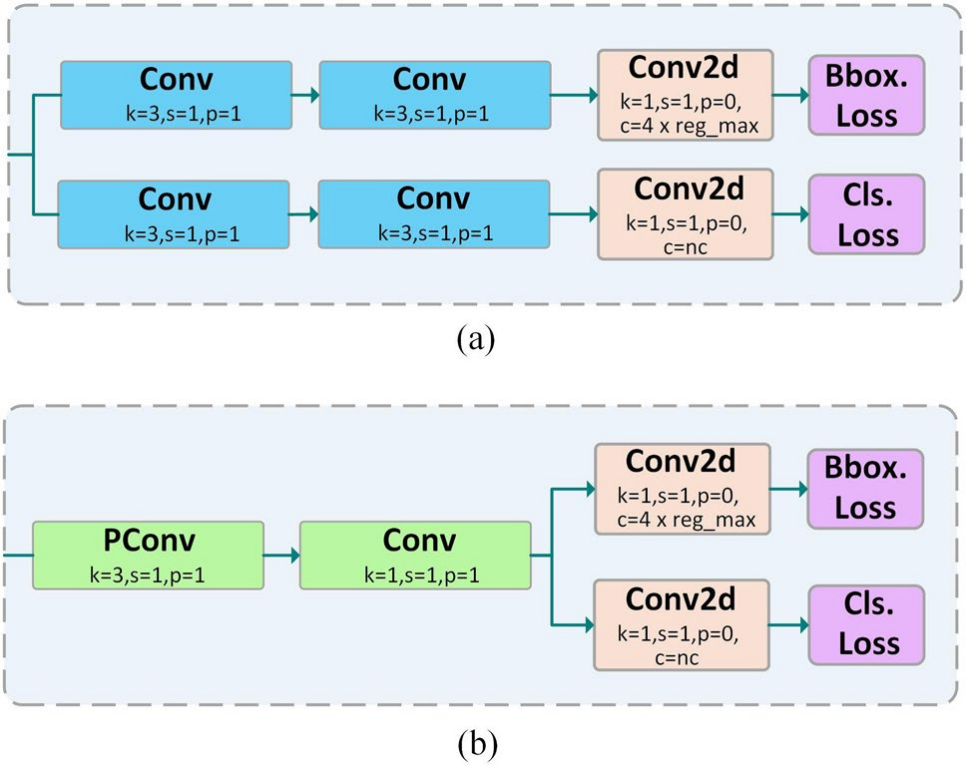
Comparison of the structure of the detection head before and after improvement.

Instead of using the coupling header structure in YOLOv5, YOLOv8 opts for decoupling headers. Each decoupling header includes two branches, one for the classification task and one for the regression task, and adopts the idea of Anchor Free. Each branch comprises two $3\times 3$ Conv layers and one $1\times 1$ Conv2D layer, so when there are more feature layers, multiple $3\times 3$ Conv layers stacked together increase the parameters and FLOPs of the head. The detection head part of YOLOv8n occupies 3.46 GFLOPs of computational complexity, which takes up 42.7% of the total computational complexity of the model. This means that the detection part occupies almost half of the total FLOPs of the model, a fact that hinders the exploration of model lightweight. As shown in Fig. 7, we were inspired by the RetrainNet^49^ detection head to propose the idea of shared parameters while balancing the accuracy of detection and model complexity. We replace the two $3\times 3$ Conv layers of the two branches in each detection head with one PConv layer and one $1\times 1$ Conv layer, which are subsequently employed for the classification and regression tasks, respectively. This partially coupled approach reduces the parameters and FLOPs while maintaining the accuracy of the detections. After the improvement, the parameters of the detection head is 0.2 M, and the computational complexity is 0.46 GFLOPs, which are 22.3% and 12.6% of the original YOLOv8n, respectively, which significantly reduces the parameters and FLOPs of the detection head, and effectively improves the model's lightness. Finally, from the point of view of model improvement, our proposed method is generalizable to arbitrary convolutions for combination, such as replacing the original structure with a combination of one $3\times 3$ Conv layer and one $1\times 1$ Conv layer or a combination of other types of convolutions.

### Improved loss function

This subsection describes the loss function design of the YOLOv8 model and our approach to optimizing the loss function. Compared with previous target detection methods, the YOLOv8 model no longer detects targets in a residual two-stage manner. It is only used for categorization detection and regression detection, which includes category loss and location loss. Concerning the category loss, the YOLOv8 model employs BCE (binary cross entropy) loss and uses the SiLU^50^ activation function. For the localization loss, the YOLOv8 model employs the CIoU loss function to compute the IoU between the detected and target frames and also employs DFL^42^ to optimize the location loss.

The CIoU loss function definition is denoted as:

4 $$\begin{array}{c}{\mathcal{L}}_{CloU}=1-IoU+\frac{{\rho }^{2}\left(b,{b}^{gt}\right)}{{c}^{2}}+\theta v\end{array}$$

5 $$\begin{array}{c}v=\frac{4}{{\pi }^{2}}{\left(\text{arctan}\frac{{w}^{gt}}{{h}^{gt}}-\text{arctan}\frac{w}{h}\right)}^{2}\end{array}$$

where $b$ denotes the predicted bounding box, ${b}^{gt}$ denotes the real bounding box. $\theta$ is a weighting function, $c$ denotes the diagonal length of the minimum bounding box, which contains the predicted and real bounding boxes, $\rho$ denotes the computed Euclidean distance between the centers of the real bounding box and the predicted bounding box, and $v$ is used to measure the similarity in the length-to-width ratio.

The CIoU loss function not only takes into account the detected frame scale loss but also improves the length and width loss, allowing the detected frame to better match the shape of the real frame. However, the last term $v$ in the formula is not well defined, and since $v$ only reflects the distinction in aspect ratio while ignoring other aspects of similarity, this may cause the CIoU loss function to optimize the similarity in an unreasonable way, thus affecting the model's performance to effectively reduce the true difference between $(w,h$*)* and $({w}^{gt},{h}^{gt})$. To solve this problem, this study draws on the approach of α-IoU^51^ to optimize the loss function. α-IoU is an IoU Loss based on the Box-Cox transform, which generalizes the existing IoU-based Loss to an innovative family of Losses termed Power IoU, comprising a power IoU component and a supplementary power regularization term. α-IoU increases the accuracy of bounding box regression by adaptive reweighting and is suitable for lightweight models for better detection.

The changed formula is shown below:

6 $$\begin{array}{c}{\mathcal{L}}_{\text{IoU}}=1-IoU\Rightarrow {\mathcal{L}}_{\alpha -\text{IoU}}=1-Io{U}^{\alpha }\end{array}$$

7 $$\begin{array}{c}{\mathcal{L}}_{\alpha -CIoU}=1-Io{U}^{\alpha }+\frac{{\rho }^{2\alpha }\left(b,{b}^{gt}\right)}{{c}^{2\alpha }}+(\beta v{)}^{\alpha }\end{array}$$

where α is a hyperparameter that can be tuned for the detector to be more agile to deal with variable levels of Bbox regression precision. According to the authors' suggestion, target detection performs better when α = 3. In this study, we name this optimized loss function as α-CIoU.

### Training set‑up

The experimental operating system used in this study is Ubuntu 18.04, while PyTorch serves as the underlying framework for the developed deep learning model. The detailed environment is shown in Table 3. The hyperparameters of the model training phase include input image size is $640\times 640$, the batch size is 64, the optimizer is SGD stochastic gradient descent, and the number of training rounds is 300 epochs. The learning rate is initialized to 0.01, with momentum and weight decay values set at 0.937 and 0.0005, respectively. All other training parameters use the default values of the YOLOv8n model. In addition, the official pre-training weight files provided in this study were used to enhance the generalization of the trained model.

**Table 3:** Environment setup.

Category	Configuration
CPU	Intel^®^ Core™ i7-13700 K 16C 24 T@5.40 GHz
GPU	NVIDIA GeForce RTX 4090 24G
System environment	Ubuntu 18.04
Torch version	2.0.1 + cu117
Programming language	Python 3.9.16

### Evaluation indicators

To thoroughly and impartially evaluate the improved model's performance, this study employs Precision, Recall, mAP, model size, parameters (Params), FLOPs, and frames per second (FPS).

TP denotes true correct (count of Color-changing melons correctly detected as the positive category), FP denotes false positive (count of Color-changing melons falsely detected as the positive category), and FN denotes missed negative (count of Color-changing melons actually belonging to the positive category but incorrectly detected as the negative category).

P denotes the ratio of correctly detected targets in the positive category to the total count of targets predicted by the model belonging to the positive category, defined as:

8 $$\begin{array}{c}{\text{P}}{\text{r}}{\text{e}}{\text{c}}{\text{i}}{\text{s}}{\text{i}}{\text{o}}{\text{n}}=\frac{TP}{TP+FP}\end{array}$$

R denotes the ratio of correctly predicted targets in the positive category to the total count of targets in the total count of real positive categories, defined as:

9 $$\begin{array}{c}{\text{R}}{\text{e}}{\text{c}}{\text{a}}{\text{l}}{\text{l}}=\frac{TP}{TP+FN}\end{array}$$

AP calculates the average precision of a single category under various recall ratios, defined as:

10 $$\begin{array}{c}AP={\int }_{0}^{1} P\left(R\right)dR\end{array}$$

The mAP is a comprehensive measure of the ability of the target detection model to perform with multiple categories, which takes the average of all the category APs, defined as:

11 $$\begin{array}{c}mAP=\frac{1}{N}\sum_{i=1}^{N} AP\end{array}$$

Higher mAP values indicate better recognition performance of the algorithm. mAP@0.5 indicates the IoU threshold is the average of the APs for each category at 0.5. mAP@0.5:0.95 indicates that the compute IoU threshold ranges from 0.5 to within 0.95 in steps of 0.05, the average of the APs for each category. The mAP@0.5 and mAP@0.5:0.95 are defined as follows, respectively :

12 $$\begin{array}{c}mAP@0.5=\frac{1}{N}\sum_{i=1}^{N} A{P}_{i}\end{array}$$

13 $$\begin{array}{c}mAP@0.5:0.95=\frac{1}{N}\sum_{i=1}^{N} \sum_{j} A{P}_{i} \left(j=\text{0.5,0.55,0.6},\dots ,0.95\right)\end{array}$$

In terms of model complexity, we used model size, parameters, and floating point operations^52^ as performance metrics for the model. Params are the overall count of trainable parameters in the model, FLOPs represent the quantity of floating point operations, and model size indicates the memory usage. The smaller these three parameters are, the simpler the model structure is, i.e., the more lightweight it is.

For detection speed, we use FPS to assess the real detection speed of the algorithm, defined as:

14 $$\begin{array}{c}FPS=\frac{1}{{t}_{preprocess}+{t}_{\text{inf}erence}+{t}_{NMS}}\end{array}$$

From the above equation, the reciprocal sum of preprocessing, inference, and non-maximal suppression (NMS) consumption time is defined as FPS, and a larger FPS value illustrates the superior real-time detection ability of the model.

## Results and analysis

**Figure 8:**
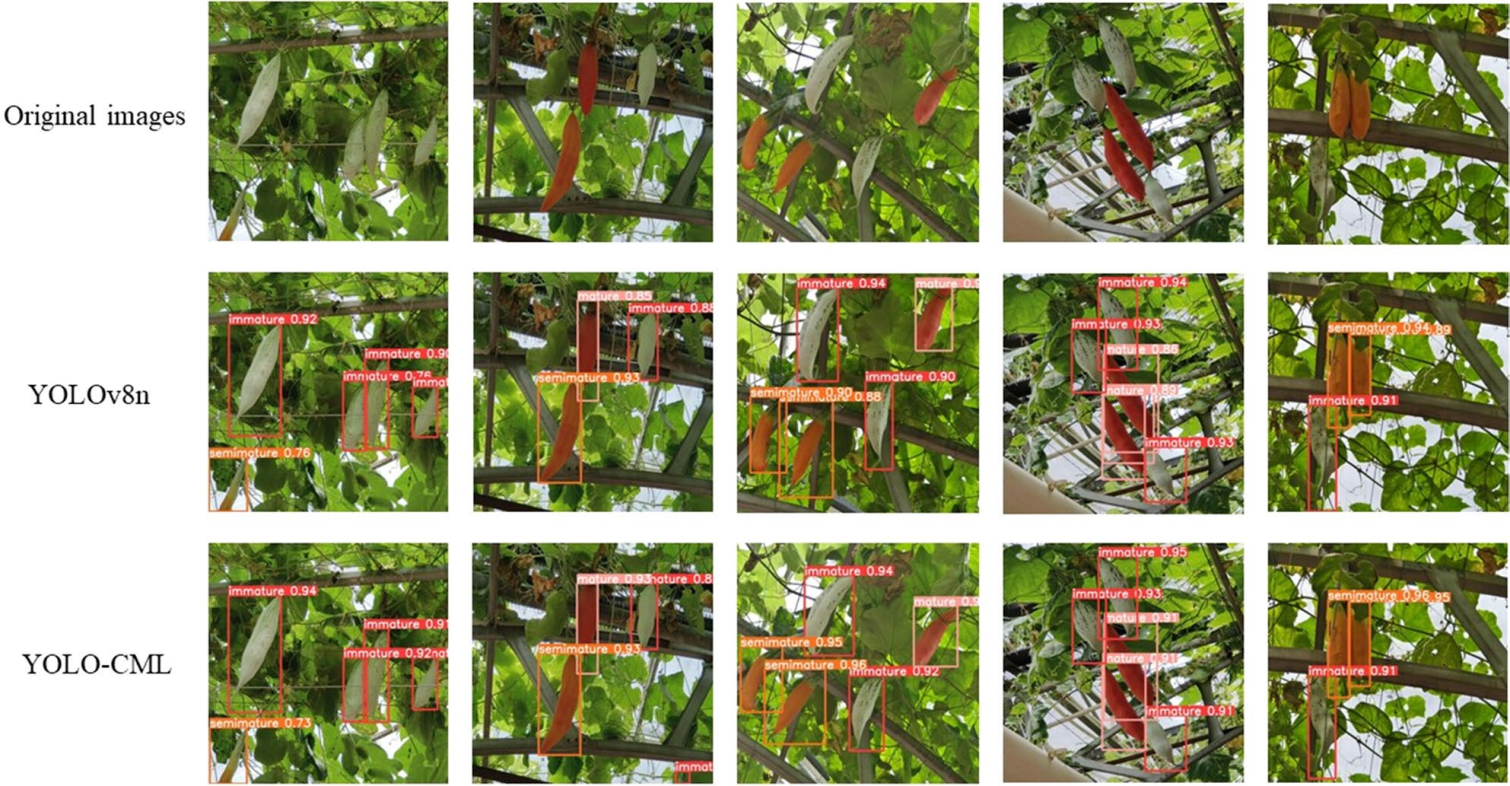
Comparison of recognition effect original and final improvement.

After the model was trained, we tested it on the test set. To assess the effectiveness of the model improvements, we compared the original and final improvements in detecting overlapping fruits, fruits occluded by leaves, and mature fruits with similar colors to the background in Fig. 8.

Compared to the original YOLOv8n, the YOLOv8-CML model uses cross-space multiscale attention to improve the detection confidence against the leaf background in the case of the many green immature fruits in Figs. 1 and 2. In the case of denser fruit in Figs. 3 and 4, the YOLOv8-CML model improves the confidence of semi-mature and mature fruit with relatively low confidence in the case of overlapping fruits in Fig. 5 the YOLOv8-CML model improves the confidence level for the occluded fruits.

Overall, the YOLOv8-CML model fluctuates in confidence for semi-mature fruits, which is related to the lightweight of the network structure. As the number of parameters decreases, the parameters related to semi-mature fruits decrease, but this has little effect on the detection accuracy in regular scenarios. It significantly improves fruit overlap and background interference scenarios.

**Figure 9:**
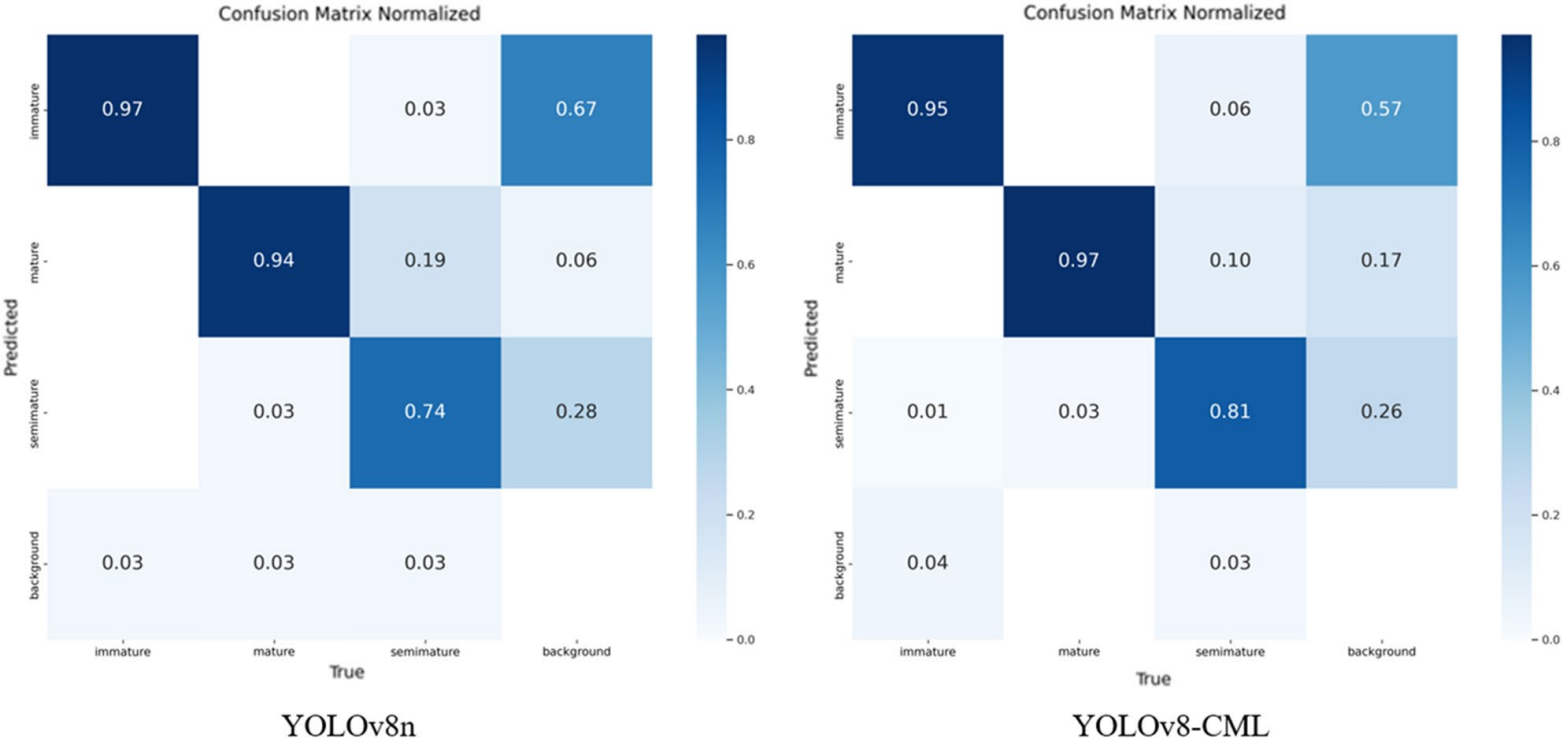
Comparison of confusion matrices before and after improvement.

Figure 9 compares the confusion matrices between the YOLOv8n model and our proposed YOLOv8-CML model. It depicts the accuracy of detecting fruit ripeness for the three categories in the dataset. In the confusion matrix, the rows represent the model's predicted labels for each class of instances, the columns represent the true labels for each class of instance labels, and the values on the diagonal represent the correct detection rate. Dark colors denote high rates, and light colors denote low rates. This visualization facilitates the observation of distinctions among instances belonging to various categories. As can be seen, our model features a comparatively high total number of values on the diagonal and reduces the false detections caused by the similarity of immature fruits with the background features while improving the detection accuracy for semi-ripe fruits.

**Figure 10:**
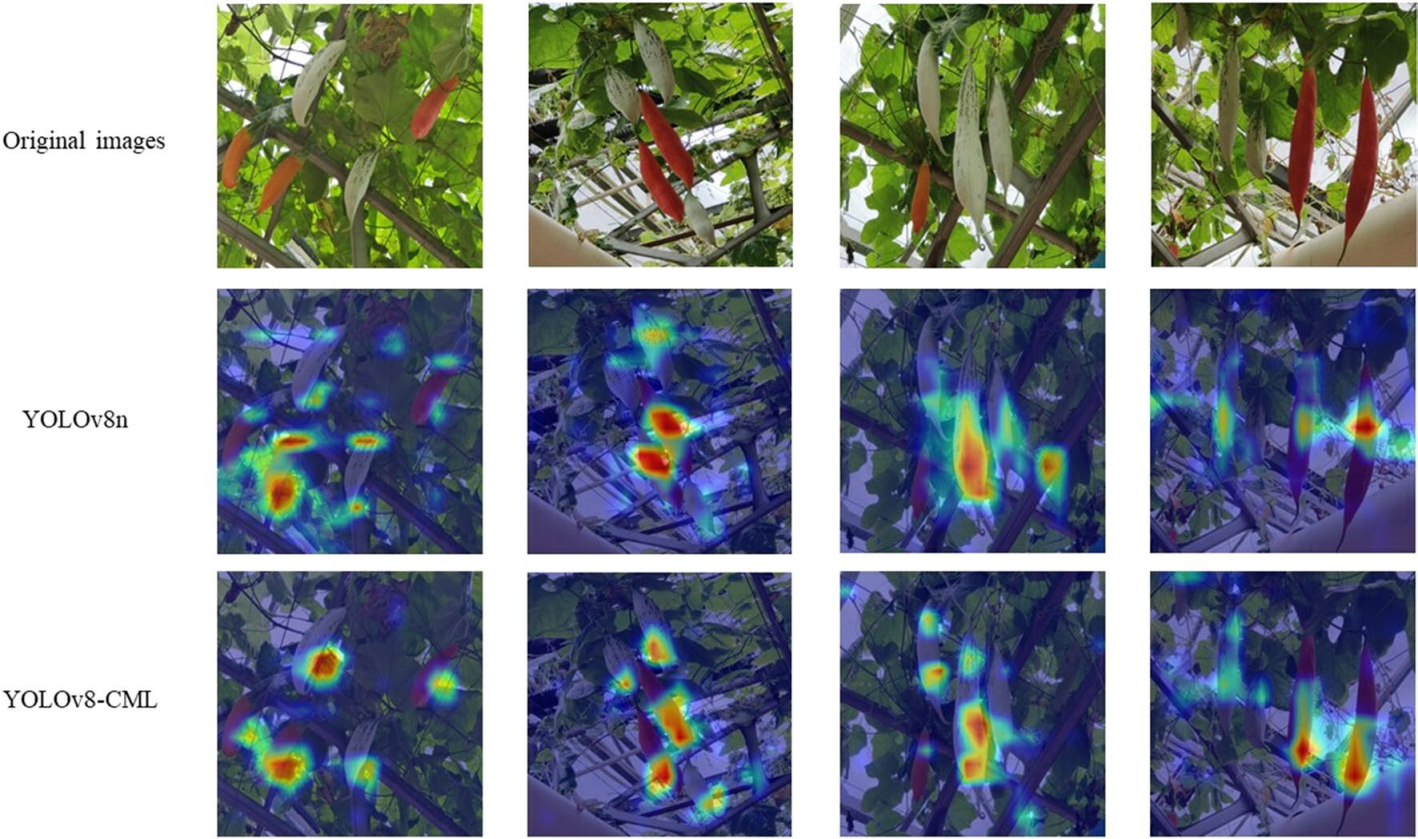
Visualization of heatmaps original and final improvements.

To visually show the effect of the improved model, we plot the hotspots of interest for the two models in two common scenarios, more dispersed fruit, and more concentrated fruit, in Fig. 10. Warmer-colored regions in the heatmap represent regions where the model is convinced of the presence of Color-changing melons. On the contrary, regions with colder colors represent regions in which the model is not confident in its prediction. It is worth noting that regions with lower heat maps do not necessarily mean that these regions do not contain Color-changing melons and that Color-changing melons may still sometimes be present in these lower confidence regions, depending on how well the features of these regions match the features of Color-changing melons that the model has learned. In Figs. 1 and 4, YOLOv8n's hotspots are more dispersed in the case of more dispersed fruit. In contrast, the hotspots of YOLOv8-CML were uniformly distributed in the fruits. In Figs. 2 and 3, the fruits are relatively concentrated, but YOLOv8n's hotspots are more concentrated in the fruits and pay less attention to the bordered fruits, while YOLOv8-CML's hotspots are more evenly distributed. This is also a side note that the efficient multi-scale attention aggregates multi-dimensional spatial information and enhances the model's ability to extract features.

To assess the improved algorithm's effect, we designed six sets of ablation experiments, which were used to compare the effectiveness of various improvement strategies on the model performance. We tested using the same devices and dataset, setting the IoU threshold to the default 0.65 in the test and keeping the other hyperparameters at the same default values to ensure comparable results. Table 4 demonstrates the outcomes of the ablation experiments.

**Table 4:** Ablation experiment results.

A	B	C	D	mAP@0.5 (%)	mAP@0.5:0.95 (%)	Precision (%)	Recall (%)	Params	GFLOPs	Size
				94.1	81.2	95.7	99	3.01 M	8.1	6.2 MB
√				92.4	78.8	94.7	97	2.30 M	6.3	4.8 MB
	√			94.5	79.0	98.5	98	2.31 M	6.4	4.9 MB
		√		94.2	79.6	97.7	99	2.42 M	5.5	5.1 MB
			√	94.4	79.3	97.5	99	3.01 M	8.1	6.2 MB
√	√	√	√	95.5	80.6	98.1	99	1.72 M	3.9	3.7 MB

A indicates using the C2f- PConv module, B indicates using the EMA-faster module, C indicates using Efficient Detection, and D indicates using α-CIoU.

First, we use the C2f-PConv module instead of the original C2f module to reduce the model's parameters and FLOPs. The improved model slightly decreases mAP and accuracy, but its complexity is also reduced. Secondly, we replaced the C2f-PConv in the Backbone part with EMA-Faster to improve the algorithm's recognition ability. Although the parameters and FLOPs have only slightly changed, the model's recognition performance has experienced a notable improvement. Next, we use only Efficient detection to decrease the model's parameters and FLOPs. We can observe a significant decrease in these parameters and FLOPs.

Furthermore, we adjust the accuracy of model detection by modifying only IoU. The α-CIoU improved the model's detection accuracy by about 2% while keeping mAP unchanged. Finally, we combine all the improvement methods to achieve the optimal balancing effect. The results showed that mAP@0.5:0.95 decreased by 0.6% and mAP@0.5 and Precision improved by 1.4% and 2.4%, respectively. The parameters and FLOPs of the YOLOv8-CML model are significantly decreased compared to the YOLOv8n model by 1.29 M and 4.2 GFLOPs, respectively.

The ablation experiments show that mAP@0.5 achieves satisfactory results by modifying different algorithm sections. However, the improved algorithm's recognition ability shows a marginal decrease compared to the standard model under stricter evaluation criteria. Overall, the proposed modifications to the original model are effective because they significantly reduce the algorithm's complexity while maintaining its central performance, thus improving its efficiency and portability.

Convolution is one of the key components in Convolutional Neural Networks. Different Conv structures have different functions, but all are essentially used to extract features. To compare the performance of various convolutional methods, we reconfigured them into the form of convolutional blocks and then tested them on an experimental platform. We first run each Conv block 1000 times in order to let the GPU "warm up," fully utilize its performance, and reduce the error caused by the difference in hardware power in the comparison process. This part of the data is not counted in the final results. Then, we immediately continue to run the Conv block 3,000 times to count its various indexes, including running time, parameters, and FLOPs. The convolutions involved in the comparison include Conv2D, DWconv2D (Depth Separable Convolution)^53^, GSConv2D^54^, DSConv2D (Distributed Offset Convolution)^55^, PConv2D (Fast Convolution)^44^, DCNV2 (Deformable Convolution) and DCNV3 (Deformable Convolution)^56^. Table 5 shows the comparison results of the various convolutions. We can observe that the best overall result is PConv2D with a mean time of 2.25 ms, followed by DWConv2D, which also works very well with a mean time of 3.35 ms, and the worst result is the ordinary Convolution Conv2D. PConv2D is not only fast but also has the least mean time. It is worth mentioning that in most cases, PConv is not used on its own. It is combined with other Convs and embedded in the model by composing a Block form, e.g., Faster Block. Given the excellent performance of PConv, we chose to use it as a core component of the improved model for the C2f module. For the same reason, we tried combinations of different kinds of convolutions in the detection head, and after comparing them in several experiments, we finally chose the combination of PConv and Conv.

**Table 5:** Comparison results for different Convs.

Name	All time	Mean time	FPS	Params	FLOPs
Conv2D	20.79834 s	6.93 ms	144.242	147.840 K	77.578 G
DWConv2D	10.06140 s	3.35 ms	298.169	18.304 K	9.731 G
GSConv2D	20.51675 s	6.84 ms	146.221	75.712 K	39.762 G
DSConv2D	19.89061 s	6.63 ms	150.824	256.000 B	268.435 M
PConv2D	7.64093 s	2.25 ms	392.622	9.472 K	5.100 G
DCNV2	44.41165 s	14.80 ms	67.567	31.387 K	16.576 G
PConv2D + Conv2D	9.14812s	3.05ms	309.483	55.679 K	30.142 G

To objectively assess the overall performance of the improved algorithm, we compared our proposed method with the present lightweight detection algorithms using the same experimental circumstances with the metrics of Precision, Recall, mAP, model size, parameters (Params), FLOPs, and FPS. The experimental results are summarized in Table 6.

**Table 6:** Comparative results of different lightweight algorithms.

Model	mAP@0.5 (%)	mAP@0.5:0.95 (%)	Precision (%)	Recall (%)	Params	GFLOPs	FPS	Size
YOLOv3-tiny	93.8	71.2	88.7	95	8.68 M	12.9	294.1	17.3 MB
YOLOv4-tiny	94.4	74.5	93.8	93	5.88 M	16.2	277.8	22.4 MB
YOLOv5n	92.7	75.5	89.6	98	1.77 M	4.2	285.7	3.9 MB
YOLOv5s	93.5	78.6	93.8	96	7.03 M	16.0	256.4	14.5 MB
YOLOv6n	94.5	78.7	98.9	98	4.70 M	11.4	303.0	9.6 MB
YOLOv7-tiny	93.4	70.8	94.0	99	6.01 M	13.1	370.3	12.3 MB
YOLOv8n	94.1	81.2	95.7	99	3.01 M	8.1	322.5	6.2 MB
Ours	95.5	80.6	98.1	99	1.72 M	3.9	344.8	3.7 MB

Under mAP@0.5, our proposed method shows promising results compared with YOLOv3-tiny, YOLOv5n, YOLOv5s, and YOLOv7-tiny.In the range of mAP from 0.5 to 0.95, our proposed method also outperforms most lightweight detection algorithms. The YOLOv5n model is close to our proposed method regarding parameters, computational number, and model size but performs poorly regarding mAP and precision. The YOLOv5s model outperforms the YOLOv5n model in terms of some performance aspects but has a lower frame rate when detecting and relatively high model complexity. The large parameters and FLOPs of the model also limit the YOLOv6n model. YOLOv7-tiny shows high performance in detection speed, but concerning mAP and precision, it is not a proper match.

After optimization by our proposed method, the improved model’s parameters and FLOPs are significantly reduced, 42.9% and 51.8% less than the original YOLOv8n model, respectively. In addition, our improved model has the smallest size while improving in terms of mAP@0.5, accuracy, and FPS. Although our improved model is slightly deficient in mAP@0.5:0.95, it has obvious advantages in all other aspects, so the loss in this part is acceptable.

Comparative results show that our proposed method achieves a better balance between accuracy and lightweight features in the Color-changing melon detection task. This important approach lays the foundation for subsequent research.

## Discussion

Deep learning-based target detection algorithms are practical for high-precision fruit picking and disease detection tasks in agriculture, a popular method often used in smart agriculture. Fast decision-making is required while working for resource-constrained devices, such as small cameras on robotic arms and micro drones. However, the complexity of the network structure of the model makes it difficult during its actual deployment. Therefore, there is a great need to consider the balance between the real-time and lightweight nature of models in agricultural inspection tasks. This study uses a lightweight YOLOv8-CML model designed for color-changing melon maturity detection. The YOLOv8-CML model performs well compared to existing lightweight models, but this study still has limitations.

The model we design must fit into a natural Color-changing melon-picking environment to make this study practical. The figures we collected were obtained from a cell phone camera taken at an 80–120 cm distance. In contrast, the small camera of the robotic arm may be closer to the fruit, depending on where the camera is mounted on the robotic arm. The purpose of using a cell phone is to capture figures from as many different angles as possible, such as occlusion and backlighting, which may occur in real-world environments, thus enriching the Color-changing melon dataset and improving the robustness of the model. However, the difference in shooting distance may affect the model's performance. In order to solve this problem, we need to study the model further and do more testing and optimization in future research to improve its robustness and better handle color-changing melon image data from different distances and angles.

## Conclusion

In this study, we propose a lightweight detection model, YOLOv8-CML, to help automatic picking robots identify Color-changing melon fruits and deploy them to edge devices more efficiently by addressing the problems of slow detection of Color-changing melon fruits and high deployment cost in smart agricultural devices. Compared to the YOLOv8n model, the parametric and computational ratios of the YOLOv8-CML model decreased by 42.9% and 51.8%, respectively. In addition, the model size is only 3.7MB, and the inference speed is improved by 6.9%, while the mAP@0.5 and accuracy are improved by 1.4% and 2.4%, respectively. In a comprehensive comparison, our proposed method achieves a better balance between model complexity and detection accuracy, which provides some ideas for deploying Color-changing melon detection models on edge devices.

In our future work, we will further explore simplifying the model while maintaining its robustness, such as compressing the model using knowledge distillation and improving the model running speed to make it more suitable for deployment on mobile or embedded devices to provide technical support for picking robots.

## Data Availability

The datasets used and analyzed during the current study are available from the corresponding author upon request. E-mail: Z22070034@s.upc.edu.cn.

## Code availability

The code used and analysed in this study is openly accessible to all, and we make the key code for the improvement module available on github at https://github.com/Alcatrazk98/YOLOv8-CML.
